# Characterization of ENM Dynamic Dose-Dependent MOA in Lung with Respect to Immune Cells Infiltration

**DOI:** 10.3390/nano12122031

**Published:** 2022-06-13

**Authors:** Angela Serra, Giusy del Giudice, Pia Anneli Sofia Kinaret, Laura Aliisa Saarimäki, Sarah Søs Poulsen, Vittorio Fortino, Sabina Halappanavar, Ulla Vogel, Dario Greco

**Affiliations:** 1 Faculty of Medicine and Health Technology, Tampere University, 33520 Tampere, Finland; angela.serra@tuni.fi (A.S.); giusy.delgiudice@tuni.fi (G.d.G.); laura.saarimaki@tuni.fi (L.A.S.); 2 BioMediTech Institute, Tampere University, 33520 Tampere, Finland; 3 Finnish Hub for Development and Validation of Integrated Approaches (FHAIVE), 33520 Tampere, Finland; 4 Institute of Biotechnology, University of Helsinki, 00014 Helsinki, Finland; pia.kinaret@gmail.com; 5 National Research Centre for the Working Environment, 2100 Copenhagen, Denmark; spo@nfa.dk (S.S.P.); ubv@nfa.dk (U.V.); 6 Institute of Biomedicine, University of Eastern Finland, 70211 Kuopio, Finland; vittorio.fortino@uef.fi; 7 Environmental Health Science and Research Bureau, Health Canada, Ottawa, ON K1A 0K9, Canada; sabina.halappanavar@hc-sc.gc.ca

**Keywords:** engineered nanomaterials, toxicogenomics, dose-dependent, TinderMIX, bronchoalveolar lavage, multiwalled carbon nanotubes, titanium dioxide, carbon black, mechanism of action, biomarker

## Abstract

The molecular effects of exposures to engineered nanomaterials (ENMs) are still largely unknown. In classical inhalation toxicology, cell composition of bronchoalveolar lavage (BAL) is a toxicity indicator at the lung tissue level that can aid in interpreting pulmonary histological changes. Toxicogenomic approaches help characterize the mechanism of action (MOA) of ENMs by investigating the differentially expressed genes (DEG). However, dissecting which molecular mechanisms and events are directly induced by the exposure is not straightforward. It is now generally accepted that direct effects follow a monotonic dose-dependent pattern. Here, we applied an integrated modeling approach to study the MOA of four ENMs by retrieving the DEGs that also show a dynamic dose-dependent profile (dddtMOA). We further combined the information of the dddtMOA with the dose dependency of four immune cell populations derived from BAL counts. The dddtMOA analysis highlighted the specific adaptation pattern to each ENM. Furthermore, it revealed the distinct effect of the ENM physicochemical properties on the induced immune response. Finally, we report three genes dose-dependent in all the exposures and correlated with immune deregulation in the lung. The characterization of dddtMOA for ENM exposures, both for apical endpoints and molecular responses, can further promote toxicogenomic approaches in a regulatory context.

## 1. Introduction

When ENMs interact with biological systems, they induce a cascade of molecular events which are mainly dependent on their physicochemical characteristics, such as the size and the surface chemistry [1,2,3]. 

Cellular responses to ENM exposures are complex, as they are trying to counteract the induced perturbation and achieve system homeostasis. In classical inhalation toxicology, bronchoalveolar lavage (BAL) cell compositions are widely used to clarify the immunopathological phenotypes in the lung [4]. ENMs exert diverse responses both in terms of cell types recruited in the lung and in their numbers [5,6,7,8,9,10]. However, apical endpoints, such as the one derived by BAL counts, do not provide details about the underlying mechanisms and responses to distinct ENMs. Usually, each significant molecular alteration triggered by ENM exposure is considered as the MOA of that compound [11].

Microarray experiments are an effective tool for the characterization of the mechanism of action detected at transcriptome levels (transcriptional mechanism of action, hereafter referred to as tMOA) [12,13,14]. However, the dissection of this response in the molecular events directly triggered by the exposure to the ENM and the ones induced by the biological adaptation process is complicated. 

In risk assessment and toxicology, dose-dependent modeling is an established tool to identify points of departure (POD) that can be used as a guidance value denoting the adverse effect levels, such as benchmark dose (BMD), no observed adverse effect level (NOAEL), and lowest observed adverse effect level (LOAEL) [15]. Similar modeling strategies have emerged for the analysis of transcriptomic experiments to identify the genes directly and progressively affected with respect to the reference dose or concentration [16,17,18,19] and to characterize their POD. 

The tMOA of an exposure arises from different mechanisms of transcriptional regulation. Therefore, some of these events are observed as dose-dependent, whereas others are consequences of more complex regulatory loops.

The common strategy for the investigation of dose responses is to study their alteration at each time point of exposure separately. However, this strategy might not accurately interpret the kinetics of the molecular adaptation processes [20,21]. The joint dose-time dynamic POD (dPOD) allows us to identify the lowest dose and earliest time points at which a notable change with respect to the controls takes place, and this effect follows a monotonic dose-dependent trend. Moreover, few efforts have been made to link the traditional immune cell quantification and transcriptional responses together [22,23,24,25]. To the best of our knowledge, the correlation between specific portions of dddtMOA and different immune cells has never been systematically investigated. 

To overcome these problems, we applied a computational approach [21] to model the dose- and time-dependent alteration patterns of publicly available BAL cell counts and lung transcriptomic responses from multiple ENMs, namely, carbon black (CB), titanium dioxide (TiO_2_), and two multiwalled carbon nanotubes (MWCNT), at three different time points. Our goal was to characterize the dddtMOA as the portion of the adaptation response directly linked to the ENM, and to further associate its effect with the immune cell infiltrations.

Our approach suggests that the dddtMOA has the potential to be applied in the context of an ENM hazard assessment, further facilitating the implementation of toxicogenomic-based approaches in a regulatory context. 

## 2. Materials and Methods

### 2.1. Dataset Collection and Preparation

Microarray-based transcriptomics data from lungs of mice exposed, by a single intratracheal instillation, to carbon black (CB), multiwalled carbon nanotubes (MWCNTs) and titanium dioxide nanoparticles were downloaded from the GEO database. Details on the nanoparticles are reported in Table 1. 

Data for MWCNTs came from the studies of Poulsen et al. [26] (available on GEO with ID GSE55286), where female C57BL/6 mice were exposed by a single intratracheal instillation to 18, 54, or 162 g/mouse of a short MWCNT (NRCWE-26, 847 ± 102 nm in length) or long MWCNT (NM-401, 4048 ± 336 nm in length). Data for CB nanomaterials came from the studies of Bourdon et al. [27,28] (available on GEO with ID GSE35193), where female C57BL/6 mice were exposed by a single intratracheal instillation to 18, 54, or 162 g/mouse of Printex 90 carbon-black nanoparticles. Data for nano-TiO_2_ nanomaterials came from the studies of Husain et al. [29] and Saber et al. [30] (available on GEO with ID GSE41041), where female C57BL/6 mice were exposed to rutile nano-TiO_2_ (primary size of 20.6 nm and surface area of 107.7 m^2^/g). Experiments related to CB and TiO_2_ were performed by using the Agilent-014868 Whole Mouse Genome Microarray 4x44K G4122F platform, whereas for the MWCNT data, the Agilent-028005 SurePrint G3 Mouse GE 8x60K Microarray platform was used. Raw mRNA expression values were imported in R v. 3.4.4 using the Limma read.maimages function [31]. Raw data were preprocessed using the eUTOPIA application [32]. First, low-quality probes were removed: only probes with a value higher than 75% quantile of negative control probes in at least 75% of the samples were selected. Afterwards, the expression values were log2 transformed and quantile normalized between arrays. Surrogate technical variables were identified and removed in the CB and TiO_2_ datasets by using the ComBat method from the R Ensembl gene ID level using annotation files provided by the microarray manufacturer (Agilent Technologies, Santa Clara, CA, USA). 

Finally, differential expression between sample groups was evaluated with the Limma package using the corrected batches as covariates for the model. The combination of the three exposure doses (18, 54, and 162 µg) and three time points (1, 3, and 28 days) yields a total of nine comparisons when each dose- and time-point combination is compared to its corresponding control sample. Genes were considered significantly differentially expressed with an absolute log2 fold change > 0.58 and Benjamini–Hochberg-adjusted *p*-value < 0.05. For the sake of comparison with respect to the dynamic dose-dependent genes, we computed the union of all the differentially expressed genes revealed by the nine comparisons and used that to characterize each ENM. 

Moreover, the BAL counts for neutrophils, eosinophils, macrophages, and lymphocytes, present in the lungs after exposure to the same ENMs considered in this study, were retrieved from previous publications [26,28,30] (Table S1).

### 2.2. Integrated Time and Dose Analysis of MOA

Sample-wise log_2_ fold changes were calculated between each exposed sample and each of its corresponding control samples. Afterwards, each gene was analyzed utilizing the TinderMIX tool [21]. Briefly, linear- and second-order polynomial models were applied for the fitting of the log_2_ fold change. For each gene, the optimal model was selected based on the lowest goodness-of-fit *p*-value. Genes with goodness-of-fit *p*-values > 0.05 were removed from the analysis. The dose and time ranges were divided into 50 equally distributed bins, and the optimal model of each gene was used to predict their corresponding log_2_ fold changes. Thus, the gene was represented by an activation map that interpolates the space of time and dose also for the experimental conditions (doses and time) not originally included in the analysis.

From each activation map, a monotonically increasing or decreasing area with respect to the dose, with a fold change greater than the activity threshold (fold change > |1.5|), was identified by analyzing the gradient vector field of the map. If present, this area is marked as responsive, and the gene is deemed altered in a dynamic dose-dependent manner. 

Eventually, an activity label was assigned to each gene by dividing both axes of the activation map into three sections and obtaining a grid with 9 cells. The sections of the dose axis were named as “sensitive”, “intermediate”, and “resilient”, whereas for time axis, labels “early”, “middle”, and “late” were assigned. The final dPOD is obtained by identifying the earliest and most sensitive activation and is labeled concatenating the corresponding dose and time labels. 

### 2.3. Integrated Time and Dose Analysis of Cell Counts

For each pair of dose and time points, the cell counts were normalized for the counts in the control sample in the following way:

(1) $$CC_{tp_{}x\;ds}=\log2\left(counts_{tr}/\;counts_{ctrl}\right)$$

where *tp* is the time point, *ds* is the dose, *tr* is the treated samples, and *ctrl* are the controls. Within the samples and controls of a particular pair of dose and time points, all the possible pairwise comparisons are performed.

Afterwards, the TinderMIX tool was used to identify the dynamic dose dependency of the BAL cell counts. Starting from the normalized cell counts, the linear- and second-order polynomial models were fitted in the joint dose-time space. The optimal model was used to predict an activation map associated with each cell count in the form of contour plots. The optimal model was identified as the one with the lowest goodness-of-fit *p*-value, among those with *p*-values < 0.05. If none of the models met the requirement for the *p*-value, the optimal model was selected as the one with the smallest AIC (Akaike information criterion). Eventually, a dPOD activity label was associated with each cell count in each material.

### 2.4. Correlation between Gene Expression and Cell Counts

For each material, the correlation between the maps of its dynamic dose-dependent genes and the maps of the cell counts were computed. For each pair of gene and cell types, we divided the map in three different regions based on if both gene and cell were dose-dependent (called region A), only one of the two was dose-dependent (called region B), or none of the two were dose-dependent (called region C). For each portion of the map, we computed a binary vector indicating if the activation was higher (1) or lower (0) than the mean activation value of that portion. The Pearson correlations between the binary vectors were computed. This led to three correlation values for each pair of gene and cell, which were combined by a weighted sum that accounted for 70% of the correlation from region A, 20% of correlation from region B, and 10% of correlation from region C. Since the aim of this study was to identify dynamic-dose dependent alterations induced by the exposure to ENMs, the weighted schema was selected to give more relevance to the area where both the gene and the cell counts were dynamic-dose dependent. However, rather than discarding correlations outside this area, they were considered still relevant to explain interactions between the genes and cell populations. To avoid spurious correlations, gene activity and cell counting maps were considered correlated when the final absolute Pearson correlation value was greater than 0.6. 

### 2.5. Pathway Analyses

The KEGG pathway enrichment analysis was performed with the compareCluster function from the clusterProfiler R package [33]. To compute the combined *p*-values for the pathways shared across the ENMs, we used the sum of logs method (or Fisher’s method) implemented in the sumlog function of the metap R package [34]. 

## 3. Results and Discussion 

### 3.1. Characterization of the dddtMOA

The tMOA of an exposure is commonly defined as the set of significant variations in gene expression observed between the exposed and unexposed samples. Therefore, we first performed a classical differential expression analysis of the dataset including exposure to TiO_2_, CB, and two MWCNTs of different length with multiple doses (18, 54, and 162 mg) and multiple time points (1, 3, and 28 days).

Particularly, we identified lists of differentially expressed genes for each combination of material, dose, and time. As for the lung inflammation, we retrieved information on BAL counts of macrophages, neutrophils, eosinophils, and lymphocytes in the same experimental conditions [26,28,30] (Table S1).

**Figure 1:**
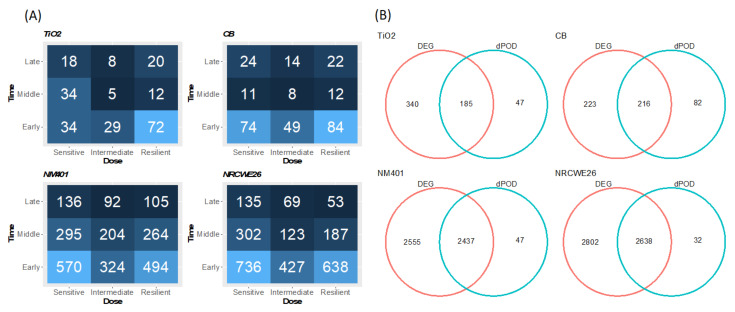
(**A**) Number of dose-dependent genes for each ENM for each of the dose-time labels. The labels can be interpreted as follows: sensitive—genes that respond at low doses; intermediate—genes that respond at intermediate doses; resilient—genes that respond at high doses.; early—genes that respond at short time points; middle—genes that respond at intermediate time points; late—genes that respond at late time points. The blue color gradient describes the number of dose-dependent genes: dark blue represents a smaller number of genes identified, whereas light blue represents a higher number of dose-dependent genes. (**B**) Number of DEG (red) and genes with a dPOD activity label (green) for each ENM.

To characterize the dynamic dose-dependent portion of the tMOA (dddtMOA) of each nanomaterial, we performed an integrated dose and time analysis of the molecular alteration induced on the whole transcriptome and we investigated the distribution of the number of dynamic dose-dependent genes across their dose and time dPOD (Figure 1A, Tables S2–S5). 

Both MWCNTs showed the highest number of dose-dependent genes (2484 and 2670 for NM401 and NRCWE26, respectively), whereas the number of genes altered by CB and TiO_2_ was lower (232 and 298, respectively). Our results suggest that the magnitude of the response could reflect the ENM hazard level. For example, a high aspect ratio and rigid nanomaterials, such as NM401, are usually more persistent in the lung, and their reduced clearance contributes to the toxic profile [35]. 

In our dose- and time-dependent analysis, the genes were labeled based on where their expression deregulation starts with respect to the time (early, middle, or late) and the dose levels (sensitive, intermediate, or resilient). 

As expected, the kinetics of the molecular response in all ENMs immediately start at short time points, having an early dPOD. This might be partially influenced by the single-exposure experimental design, where stronger acute responses can be detected at shorter time intervals, whereas mechanisms associated with recovery can be detected at later time points. However, our results are consistent with the acute adaptive response to ENM exposure [36]. As for the exposure dose, MWCNTs seem to activate most genes at low doses, having a sensitive dPOD (Figure 1A). As discussed before, the dose required to induce the alteration can be linked to the toxicity level of the ENMs. Interestingly, the most represented label in the CB and TiO_2_ exposures is early resilient. This may indicate that for spherical-shaped nanomaterials, a portion of the response requires higher doses to be evident. 

To functionally characterize the dddtMOA, we first compared the differentially expressed genes (DEG) and the genes with a dPOD activity label of the four ENMs, and we found an intersection in all the materials (Figure 1B). Particularly, 185 genes were found for the TiO_2_, 216 for the CB, and 2347 and 2638 for the NM401 and NRCWE26, respectively. This supports our assumption that the MOA of the different ENMs is only partially dose-dependent, and that monotonic portions of the molecular adaptation response may be directly altered by exposure. 

**Figure 2:**
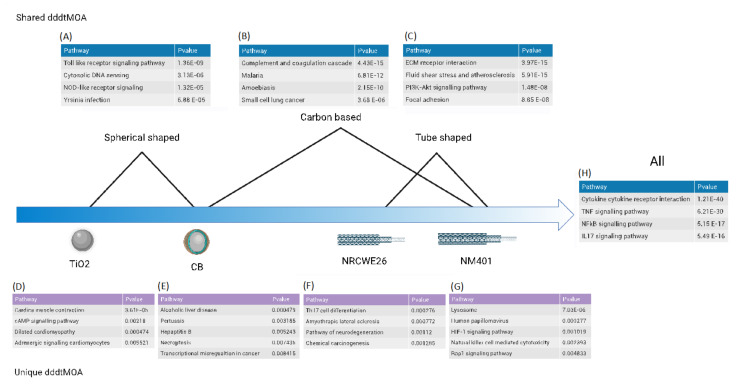
Functions of the dddtMOA shared across ENM categories (**A**,**B**,**C**,**H**) and specific to each ENM (**D**,**E**,**F**,**G**).

We then investigated the functional profile of the dPOD genes by mapping them onto the KEGG pathways (Tables S6–S9). The functionalities associated with each dddtMOA were ENM specific; however, some functions were enriched by all the studied nanomaterials, as well as by specific subcategories (Figure 2). Among the specific ENM responses, our analysis highlighted pathways associated with cardiac muscle contraction and signaling in cardiomyocytes, suggesting a possible cardiotoxic effect of titanium dioxide (Figure 2D). It was previously shown that exposure to TiO_2_ nanoparticles both in vitro and in vivo increases cardiac excitability and conduction velocity [37,38]. Similarly, the most significant alterations in NM401 exposure pointed to lysosomal effects (Figure 2G). Straight and rigid carbon nanotubes, such as NM401, are known to induce disruption of cellular structures and vesicles, including the lysosomes [6]. 

As for shared responses, all spherical-shaped ENMs enriched pathways related to microorganisms and viral infections (Figure 2A). Similarities in the host response between carbon-based ENMs and viruses were already observed [39,40]. Interestingly, pattern-recognition receptors are also commonly present. Toll-like receptors are the sentinels of foreign biomolecules, and they mediate unspecific recognition of damage- and pathogen-associated molecular patterns (DAMP and PAMP, respectively). This may suggest that the exposed cells sense spherical nanoparticles through similar signaling pathways that evolved to recognize pathogens, reinforcing the importance of physicochemical properties of the nanomaterials in shaping the adaptive response. Interestingly, toll-like receptors are known to induce necroptosis, an inflammatory form of programmed cell death, which was enriched in the carbon-black dddtMOA. MWCNTs specifically enrich pathways related to the extracellular matrix and focal adhesion, as well as vascular structures (Figure 2C). MWCNTs are known profibrotic agents, and their hazardous effects on the lung epithelium were already described in detail [40,41,42,43]. Interestingly, carbon-based materials (CNMs) share a direct effect on the complement and coagulation cascade (Figure 2B). CNMs are indeed used in coagulation disorder therapy because of their potential to interact with platelets and induce thrombosis and hemolysis [44].

Finally, all the investigated ENMs shared a core of functions related to inflammation and immunity (Figure 2H). Nanomaterials trigger the immune response and induce activation of inflammatory mediators through various mechanisms. Importantly, NFkB and TNF are very well characterized mediators of nanomaterial adaptation [45,46,47,48]. Similarly, upregulation of a plethora of cytokines was reported for all the studied nanomaterials [45,46]. 

### 3.2. Combination of Transcriptional Changes and BAL Cell Count Dose-Time Responses Informs on ENM-Specific Immune Cell Activation

The interaction of ENMs with the immune system is an accepted theory; however, clarifying the cellular mechanism of ENM-mediated toxicity is still considered a major challenge. 

We hypothesized that combining the dose-dependent and time-related transcriptional changes with the BAL cell count activation may help to better characterize the molecular basis of the immune cell activation and response.

First, we performed a similar dose- and time-integrated analysis on the BAL cell counts and identified their joint dynamic dose-dependent dPOD (Table S10). 

In the case of neutrophils, the dPOD for all the ENMs was determined as sensitive-early, specifying a dynamic dPOD starting at a low dose range and an early time interval and preserved through the postexposure time points. This suggests that the immediate cell infiltration consequent to the insult extends until later time points. Neutrophils have long been known as “the first line of defense”, as well as the initiator of the inflammatory response [49]. In this light, it is expected that neutrophils, as quickly reacting cells, are labeled as early responders. 

Macrophages showed different dPOD labels in different ENMs. They were sensitive-early in all the CNMs and intermediate-late in TiO_2_. Macrophages are equally considered as rapid responders, but unlike neutrophils, which migrate to the site of insult and survive only 1 to 2 days, macrophages are already residing in lung tissue and survive much longer at the site of inflammation. More circulating macrophages might be recruited at later time points or higher doses, thus possibly explaining their varying activation patterns across the different ENMs. 

Eosinophils showed sensitive-late dPOD in CB, still suggesting cell infiltration after the exposure but at the longest time points [50].

Lymphocytes also showed a dose-time response only in spherical-shaped ENM. In the case of CB they were labeled as sensitive-early, whereas in TiO_2_ they were activated later at low doses.

**Figure 3:**
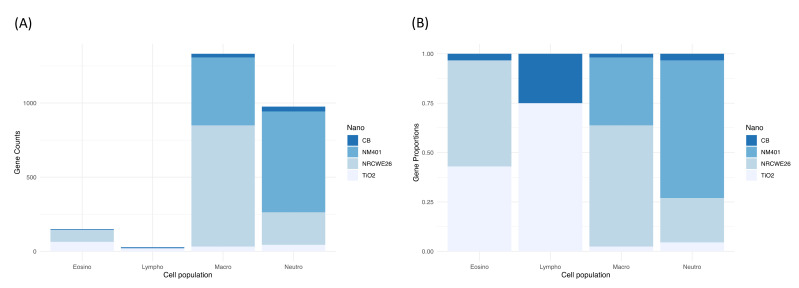
(**A**) Number of dynamic dose-dependent genes correlated with cell counts for each ENM. (**B**) Proportion of dynamic dose-dependent genes correlated with cell counts for each ENM.

To contextualize the dddtMOA with respect to the activation of immune cells, we identified the sets of genes with a dynamic dose-dependent activation which were also correlated with the immune cell counts (Figure 3).

Macrophages and neutrophils showed the highest number of correlated dPOD genes (Figure 3A). This can be easily explained by their key role in acute responses to exogenous compounds, as well as their extremely plastic activation upon cues received from their immediate microenvironment [51,52]. 

However, when we analyzed the relative composition, a difference between nanotubes and spherical-shaped nanomaterials arose (Figure 3B). MWCNTs are predominantly correlated with macrophages (about 75% for NM401) and neutrophils (about 60% for NRCWE26). On the contrary, TiO_2_ mainly induced the response of lymphocytes and eosinophils, and CB of lymphocytes exclusively. Several studies have already shown that ENMs can interact in diverse ways with the immune system [36]. The difference seems to be driven by physicochemical characteristics, such as the shape and the size, or the hydrophobicity level. For example, Liu et al. [53] proved that the diameter in silica–titania nanoparticles directly affects the amplitude of the inflammatory reaction. 

In conclusion, correlating the dddtMOA transcriptional profile with dPOD labels derived by cell BAL counts can inform on different cellular mechanisms induced by specific ENMs. 

#### 3.2.1. ENM Physicochemical Properties Induce Differences in Immune Activation

**Figure 4:**
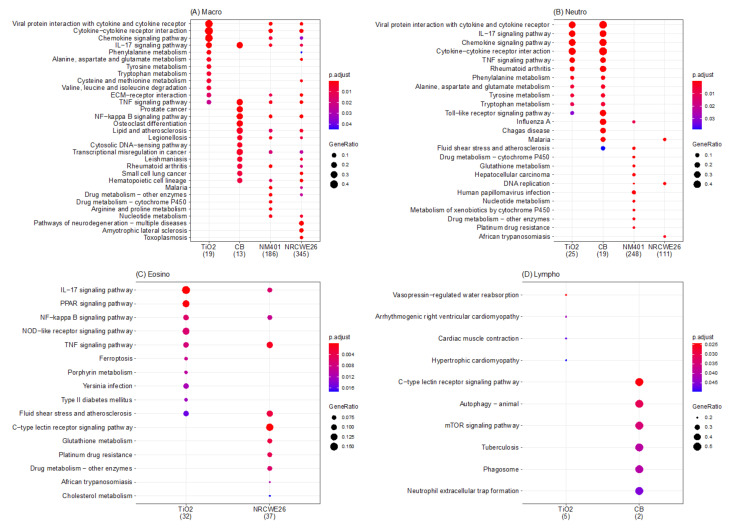
KEGG pathways of the dynamic dose-dependent genes for the different ENMs. The different panels represent the pathways enriched by the dose-dependent genes that for each ENM correlate with macrophages (**A**), neutrophils (**B**), eosinophils (**C**), and lymphocytes (**D**). The size of the dots represent the gene ration whereas the color represent the adjusted *p*-values of the enrichment analysis. The numbers in brackets below the ENM names represent the number of genes correlated with the cell populations.

To characterize the dynamics of the immune response for each ENM, their individual functional profiles were investigated by mapping the genes onto the KEGG pathways (Figure 4, Tables S11–S14). Two clear patterns emerged from genes correlated with neutrophils and macrophages. 

**Figure 5:**
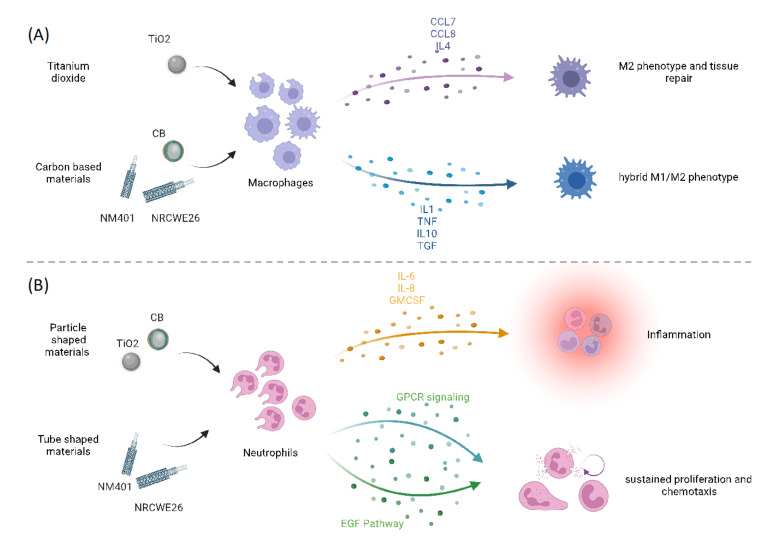
Graphic representation of the effect of ENM psychochemical properties on immune cell populations. In (**A**), the effect of different-shaped ENMs on neutrophils is reported by showing the different mediators and signaling pathways activated. In (**B**), ENMs with different chemistry are shown to differently polarize macrophages.

Our set of macrophage-correlated genes (Figure 4A) in TiO_2_ nanoparticles include several mediators, such as CCL7, CCL8, CCL9, and IL4. IL4 is a powerful inducer of M2 polarization and marker of M2 macrophages. M2 macrophages are known to promote tissue repair and an anti-inflammatory phenotype [54]. Comparable results were previously observed for TiO_2_ nanotubes, which exert a protective effect on epithelial integrity by inducing M2 macrophages [55,56]. CNMs, instead, even if with different amplitudes, share a common functional core of the macrophage adaptive response, including the expression of IL1, NfkBia, TNF, IL10, and TGF. Both TNF and IL1 belong to the panel of inducers of M1 polarization, whereas IL10 and TGF belong to the M2 panel instead. We previously described that CNMs trigger macrophages towards a hybrid M1/M2 phenotype in a nonacute exposure setup [46]. This evidence points to a different mechanism of polarization of macrophages across ENMs that is possibly dependent on ENM chemistry (Figure 5A).

As for neutrophil-correlated genes (Figure 4B), they were characterized by a different adaptive response in TiO_2_ and CB as compared to NM401 and NRCWE26. 

Genes correlated with neutrophils in TiO_2_ and CB were monocyte-lineage inducers and chemoattractants related to chemokines and cytokine receptor–ligand activity (Figure 4B). It is well recognized that the successful initiation and resolution of inflammation is mediated by neutrophils. GM-CSF genes, such as Csf2 and IL8, directly promote proliferation and maturation of neutrophils, but also affect macrophages and eosinophils via STAT3 and STAT5, facilitating defense against infections. In fact, most of these genes correlated with neutrophils overlap between the two spherical-shaped ENMs, highlighting that sustainment and homeostasis of neutrophils is a conserved mechanism between them. However, a set of genes was specific for CB, including IL6, Clec4n, CCL12, Ctsk, IL33, Pigr, Vinn1, and Ifit1. IL6 is a crucial mediator since it induces neutrophilia under inflammation. Furthermore, when activated via STAT3, IL6 signaling represents an important event for the termination of the immune response [57,58]. Overall, these results point to the central role of the JAK-STAT pathway in sustaining neutrophil proliferation and production of inflammatory mediators (Figure 5B).

On the contrary, neutrophil-correlated genes in MWCNTs suggest induced neutrophilia through a different mechanism (Figure 5B). NRCWE26 has only a few genes correlated with neutrophils, among which are genes associated with cell proliferation (such as Pol2 and Mcm2/3), and, interestingly, various hemoglobin chains and subunits. Hemoglobin was recently described to have a pro-inflammatory and pro-oxidant effect, and it has become clear that it can trigger neutrophil infiltration by recognition of DAMP or GPCR [59,60]. Multiple studies report that the main effect of neutrophils when triggered through GPCR is sustained proliferation and chemotaxis [61]. NM401 neutrophil-correlated genes enrich the epidermal growth factor (EGF) pathway and multiple pathways related to glutathione transferases. Uddin et al. demonstrated that EGF production by epithelial cells, a well-known factor of epithelial repair, increases neutrophil accumulation, which can promote intervention for acute injury, as well as potentially enhance chronic inflammation in the airways [62]. 

Overall, these results suggest that the mechanism of neutrophil activation is different across ENMs (Figure 5B). This could be due to the activation of a different panel of membrane receptors, and hence, we hypothesize the role of ENM shape as a critical property in activating a more epithelial or strictly immunological response. Supporting our hypothesis, Danielsen et al. already observed that pulmonary exposure to differently shaped CNMs induced differences in the neutrophil influx in vivo [63]. In addition, CB-induced neutrophil influx and transcription of pro-inflammatory genes in lung tissue were shown to be unaffected by TLR2 and TLR4 status, whereas CNT-induced neutrophil influx was somewhat reduced in TLR4^−/−^ mice as compared to wildtype mice [63].

Finally, genes correlated with lymphocytes and eosinophils (Figure 4C,D) enriched only a few pathways and did not show specific trends associated with ENM properties. A possible explanation could be the small number of genes correlated with eosinophils and lymphocytes. 

Of note, even if the role of eosinophils in TiO_2_ and NRCWE26 has not been investigated in detail in the community, our results highlight genes enriching the IL-17 pathway. Th17-derived cytokines, such as IL-17A and IL-17F, exert distinct effects in airway inflammation, and it was postulated that aberrant IL-17A/F production may drive severe forms of asthma [64].

#### 3.2.2. Dynamic Dose-Dependent Genes Shared by All the ENMs

Increasing evidence suggests that dose-dependent alterations highlight the most biologically significant events [65]. In this light, benchmark dose analysis can explain the MOA of ENM exposures by highlighting relevant responders already at short time points. 

**Figure 6:**
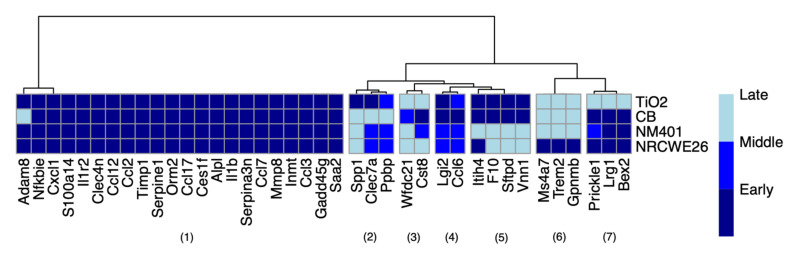
Genes with a dynamic dose-dependent transcriptomic profile in the four ENMs. Colors indicate the dynamic point of departure of the gene with respect to early, middle, and late time points.

The approach applied here revealed 39 genes with dose-dependent alteration in the four ENMs that show, however, a specific dynamic pattern for each ENM (Figure 6). 

The genes were clustered into seven groups based on their dynamic dose-dependent dPOD genes. Some clusters show specific labels for individual or grouped ENMs. Interestingly, cluster 5 shows an early alteration in the spherical-shaped nanomaterials, but not in the carbon nanotubes. The Sftpd gene in this group was previously proposed as a biomarker of TiO_2_ exposure [66]. Cluster 7, instead, shows an early alteration specific to CNMs. The Lrg1 gene in this group is an acute phase protein mainly expressed in neutrophils, involved in wound healing and fibrosis. Notably, it was proposed as a reliable marker of airway inflammation in asthma [67]. 

The biggest cluster includes genes with early activation across the four nanomaterials. Since the aim of our work was to identify dose-dependent responder genes that also correlate with the phenotype observed in the lungs, we focused our analysis on those that are also correlated to at least one cell population in each ENM. Of these, three genes met our criteria, namely, CCL12, CCL7, and IL1b (Table 2). The three genes mostly show a sensitive-early dPOD, meaning that they are already activated at low doses and early time points. However, CCL7 and CCL12 show a resilient response in TiO_2_ and CB. Moreover, the genes show a material-specific correlation with the different cell populations (Table 2). 

CCL7 is an important chemoattractant of neutrophils, macrophages, and natural killer cells, whose expression allows prompt control and the initiation of inflammation [68]. Importantly, CCL7 is a key player in the resolution of inflammation in lungs, suggesting that it may have a central role in the recovery phase of the exposure [69]. CCL12 binds to the same receptor of CCL7, and these axes seem to play a key role in insult response in the lung. In detail, mice with lung epithelial cell-specific deletion of CCL12 were protected from bleomycin-induced fibrosis and had expression of CCL2 and CCL7 similar to that of control mice treated with bleomycin. Many MWCNTs seem to induce bleomycin-like fibrosis [40]. The protective effect of low levels of CCL12 against fibrosis makes this gene an important factor and possibly a therapeutic target. IL1b is an important marker of inflammation, already established in cancer as a prognostic and therapeutic target [70,71]. Importantly, IL1 stimulates CCL7 expression through the NfkB and MAPK pathways in astrocytes [72]. Even if this relationship has not been proven in the lung, our results suggest an important cytokine–chemokine axis, whose deregulation is potentially predictive of lung toxicity.

## 4. Conclusions

Even though ENMs are widely spread, clarifying the molecular events and cellular mechanisms of adaptation upon exposure is still considered a challenge [12]. BAL cell counts are commonly used to identify inflammatory events in the lungs. Although they help identify which cell types and in which proportion they contribute to inflammation, they lack the ability to inform on the crosstalk across the different populations, as well as molecular mediators. To overcome this problem, toxicogenomic studies were successfully applied to characterize the MOA of nanomaterials. 

Disentangling the direct effects of the exposures from other secondary ones arising from complex regulatory loops is a main challenge in toxicogenomics. Here we assumed that genes whose expression is dose-dependent are directly altered by the exposure at that specific dose. Dose-dependent modeling is indeed an effective method for the identification of transcriptomic alterations that have a monotonic trend across the doses. However, classical dose-dependent analyses are performed at each time point separately, thus limiting the capability of describing the kinetic effects of the exposures. 

In this study we performed an integrative analysis to characterize the dynamic dose-dependent portion of the dddtMOA of four ENMs, namely, TiO_2_, CB, and two MWCNTs. We disentangled the dddtMOA and highlighted specific, functional profiles of each nanomaterial and subcategory. Furthermore, we modeled the commonalities between the dddtMOA and four immune cell populations. We demonstrated that dynamic dose-dependent and differentially expressed genes, that also correlate with the patterns of infiltrations of immune cells in the lungs, are effective in characterizing the direct effects of the exposures. Furthermore, our analyses highlighted specific adaptive responses modulated by different properties of the nanomaterials, such as chemistry and shape. The effect of specific properties on the mediators’ induction has important, potential therapeutic applications.

The integrated dynamic dose-dependent analysis has the potential to highlight key mediators and early responders of exposures. Here we highlighted a panel of 39 genes with dynamic dose-dependent profiles across the nanomaterials. Of these genes, three were correlated to at least one immune cell population, having the potential to inform on lung immunotoxicity.

The present study has several limitations. The recovery after acute toxic response that is highlighted by our analysis might be partially biased by the experimental setup used when performing transcriptomic experiments, which includes only one exposure at the baseline. This could also explain why most of the genes identified are correlated with macrophages and neutrophils, which are known to be early responders against intruders. The availability of a bigger set of ENMs and data from repeated exposures might help in characterizing a chronic adaptive response that would correlate with lymphocytes and eosinophils. Furthermore, the association between physicochemical properties of nanomaterials and the adaptive response may be more robustly detected by including a wider set of nanomaterials. 

Nonetheless, the definition of toxicological points of departure for key genes altered by ENM exposures opens new perspectives in the application of transcriptomic data for regulatory purposes. 

## Figures and Tables

**Table 1:** Datasets used in this study.

ENM ID	Type	Length/Diameter (nm)	Surface Area (m^2^/g)	No. Samples	Doses (µg)	Time (Day)	GSE	Ref.
NM401	Multiwalled carbon nanotube	4048/67	18	139	18/54/162	1/3/28	GSE55286	[26]
NRCWE-026	Multiwalled carbon nanotube	847/10	245	139	18/54/162	1/3/28	GSE55286	[26]
CB (Printex 90)	Carbon black	14 (diameter)	295–338	67	18/54/162	1/3/28	GSE35193	[27,28]
TiO_2_ (L181 UVTitan)	Nano-TiO_2_	20.6 (diameter)	107.7	65	18/54/162	1/3/28	GSE41041	[29,30]

**Table 2:** Genes that are dose-dependent in all the ENMs and are correlated to at least one cell population. In dPOD column: RE stands for resilient-early, SE stands for sensitive-early. In cell population column: N stands for neutrophils, M stands for macrophages, E stands for eosinophils.

Gene	Description	ENM	dPOD	Cell Population
CCL7	chemokine (C-C motif) ligand 7	TiO_2_	RE	N, M
		CB	SE	N
		NM401	SE	M
		NRCWE26	SE	N
CCL12	chemokine (C-C motif) ligand 12	TiO_2_	RE	E
		CB	RE	N
		NM401	SE	N
		NRCWE26	SE	N
IL1b	interleukin 1 beta	TiO_2_	SE	E
		CB	SE	M
		NM401	SE	M
		NRCWE26	SE	M, E

## Data Availability

Publicly available datasets were analyzed in this study. This data can be found here: https://www.ncbi.nlm.nih.gov/geo/query/acc.cgi?acc=GSE55286 (accessed on 1 December 2020); https://ncbi.nlm.nih.gov/geo/query/acc.cgi?acc=GSE35193 (accessed on 1 December 2020).

## Supplementary Materials

The following supporting information can be downloaded at: https://www.mdpi.com/article/10.3390/nano12122031/s1, Table S1: Table of BAL fluid cell data; Table S2: List of dynamic dose-dependent genes in TiO_2_; Table S3: List of dynamic dose-dependent genes in CB; Table S4: List of dynamic dose-dependent genes in NM401; Table S5: List of dynamic dose-dependent genes in NRCWE26; Table S6: List of KEGG pathways enriched by the dynamic dose-dependent genes in TiO_2_; Table S7: List of KEGG pathways enriched by the dynamic dose-dependent genes in CB; Table S8: List of KEGG pathways enriched by the dynamic dose-dependent genes in NM401; Table S9: List of KEGG pathways enriched by the dynamic dose-dependent genes in NRCWE26; Table S10: dynamic dose-dependent labels of the immune cell BAL counts; Table S11: List of KEGG pathways of the dynamic dose-dependent genes correlated with macrophages; Table S12: List of KEGG pathways of the dynamic dose-dependent genes correlated with neutrophils; Table S13: List of KEGG pathways of the dynamic dose-dependent genes correlated with eosinophils; Table S14: List of KEGG pathways of the dynamic dose-dependent genes correlated with lymphocytes.
