# Supplementary material for: Characterization of ENM Dynamic Dose-Dependent MOA in Lung with Respect to Immune Cells Infiltration
Source: Nanomaterials (Basel). 2022 Jun 13;12(12):2031. doi: 10.3390/nano12122031 (PMC9228743; doi:10.3390/nano12122031)
Supplement: Supplementary file 1 [file nanomaterials-12-02031-s001.zip › Table S10.pdf]

Supplementary Table S10. TinderMIX activation labels for the dose-time integrated model of cell counts. Abbreviations SE, IE, SM and LS signify "dose-time" labels, correspondingly. SE stands for sensitive-early; IE for intermediate-early. SM stands for sensitive-middle, and SL stands for sensitive-late. - indicates no dynamic-dose-dependent effect.

	Macrophages	Neutrophils	Eosinophils	Lymphocytes
MWCNT26	SE	SE	SE	-
MWCNT401	SE	SE	-	-
CB	SE	SE	SL	SE
TiO_2	IL	SE	SE	SL
